# Matrix Effect Variability in Urine Samples from Different Cohorts and Implications on LC-ESI-MS Mycotoxin Biomarker Analysis

**DOI:** 10.3390/toxins18030135

**Published:** 2026-03-10

**Authors:** Michael Kuhn, Åsa Svanström, Nicholas N. A. Kyei, Sanna Lignell, Hans-Ulrich Humpf, Benedikt Cramer

**Affiliations:** 1 Institute of Food Chemistry, University of Münster, Corrensstraße 45, 48149 Münster, Germany; 2 Division for Risk and Benefit Assessment, Swedish Food Agency, P.O. Box 622, 75126 Uppsala, Sweden; asa.svanstrom@slv.se (Å.S.); sanna.lignell@slv.se (S.L.); 3 Institute of Public Health, Charité–Universitätsmedizin Berlin, Corporate Member of Freie Universität Berlin and Humboldt-Universität zu Berlin, Charitéplatz 1, 10117 Berlin, Germany; 4 Research Department 2, Potsdam Institute for Climate Impact Research (PIK), Member of the Leibniz Association, P.O. Box 60 12 03, 14412 Potsdam, Germany; 5 Heidelberg Institute of Global Health, Heidelberg University, Im Neuenheimer Feld 324, 69120 Heidelberg, Germany

**Keywords:** matrix effect, LC-MS/MS, urine samples, biomonitoring, mycotoxin biomarkers

## Abstract

Matrix effects (ME) during LC-ESI-MS analysis are a commonly acknowledged issue for a variety of matrices and analytes. Although sample preparation techniques are steadily evolving to reduce ME, the complexity and variability of the urine matrix remain a challenge, especially for multi-analyte methods. To investigate the extent of ME implications on method performance and quantification, we used stable isotope-labelled standards (SIL-IS) of 11 mycotoxins to evaluate the magnitude and variability of ME in urine samples from two cohorts: Bangladeshi adult women (*n* = 50) and Swedish children of both sexes (*n* = 340). Significant ME differences were observed between the two cohorts for eight of the 11 mycotoxins. Additionally, intra-cohort ME variability turned out to be very high with interquartile ranges (IQR) above 15% for 14 out of 22 analyte-cohort combinations. Maximum IQR values were observed for sterigmatocystin in the Bangladeshi cohort (318%), strongly impacting quantitative results obtained with matrix(-matched) calibration. Further experiments on a small German cohort of four subjects, each providing four to five urine samples, revealed high variability of ME within each individual. Factors influencing ME were investigated, showing little to no impact of sex and a moderate impact of age for some analytes in the Swedish cohort. Nonetheless, especially the more polar analytes, showing stronger signal suppression, demonstrated clear correlation of ME with density and creatinine concentration of the urine samples. As a result, urine samples with very high or low density or creatinine values require careful handling in regard to sensitivity or quantification errors when matrix(-matched) calibration without SIL-IS is applied.

## 1. Introduction

Liquid chromatography coupled with electrospray ionization–tandem mass spectrometry (LC-ESI-MS/MS) is currently the technique of choice for a variety of applications requiring sensitive and selective analysis. Consequently, LC-ESI-MS/MS is widely used for human biomonitoring and exposure assessment of numerous substances in physiological samples. However, biological matrices such as plasma and urine are particularly prone to matrix effects (ME) [1,2]. These describe a change in signal response of a compound measured in the respective matrix compared to the signal in pure solvent [3]. A greater response in the matrix compared to the solvent is usually termed signal or ion enhancement, while a reduced response is called signal or ion suppression. Both are results of changes in ionization efficiency caused by matrix components co-eluting with the respective analyte. ME can thus have significant implications for the sensitivity, reproducibility, and accuracy of quantitative results [4]. In urine, often used for human biomonitoring studies, the ME can be caused by various matrix components like salts, amino acids, carbohydrates, proteins, phospholipids, and other excreted compounds [5]. Beyond the matrix itself, ME has been reported to depend on the ionization source (e.g., electrospray or chemical ionization) and the applied ionization polarity [6]. It has been suggested that the physicochemical properties of analytes play a limited role in ME, affecting only the retention time (RT) and, thus, the matrix components co-eluting with the analyte [7,8]. Different strategies exist for overcoming ME. The first approach should be effective sample clean-up, which removes unwanted matrix components and thereby reduces ME. This way, losses in sensitivity are avoided and compensation of ME becomes unnecessary. However, in a complex matrix such as urine, particularly when using multi-analyte methods, achieving sufficient clean-up can be challenging. A common approach is simple dilution of the sample, as this can reduce the ME to a negligible extent [9]. At the same time, however, sensitivity is reduced, and because no matrix is removed, interferences of the measurement may still occur. In addition to lowering ME, it can also be compensated for, e.g., by adding analyte analogues, ideally stable isotope-labelled internal standards (SIL-IS), at a known concentration, or by using matrix(-matched) calibration with a suitable blank matrix. The use of SIL-IS provides highly effective and convenient compensation but is limited by its cost and commercial availability. When SIL-IS are unavailable or too costly and standard addition is too laborious for large sample sets, matrix(-matched) calibration can be employed. This approach requires access to a sufficient analyte-free matrix that closely represents the samples being analyzed, accounting for the ME in each sample. However, this relies on a homogeneous and consistent sample composition. In highly variable matrices such as urine, deviations between the blank matrix and the actual samples can introduce significant quantification errors if the ME differs between them [4]. The International Council for Harmonisation of Technical Requirements for Pharmaceuticals for Human Use (ICH) guideline on “Bioanalytical Method Validation and Study Sample Analysis” recommends that ME be evaluated in at least six different sample sources or lots, with individual accuracy and precision within 15% (coefficient of variation) [10]. In biomonitoring studies, urine sample cohorts often comprise several hundred individual samples to represent the exposure of a specific population. Because factors such as age, sex, diet, geographic origin, and sampling time can influence urine composition and concentration [11,12,13,14,15,16], the ME may vary accordingly. To our knowledge, systematic investigations of ME variability in large urine cohorts used for biomonitoring have not been reported. Using SIL-IS, we assessed the ME in two distinct cohorts of pregnant Bangladeshi women (*n* = 50) and of Swedish children (*n* = 340), comparing overall ME and its variability. We further examined the relationship between ME and factors such as subjects’ age and sex, as well as urine creatinine concentration and density, to better understand the underlying causes of ME variability and their implications for biomonitoring studies.

## 2. Results

**Figure 1:**
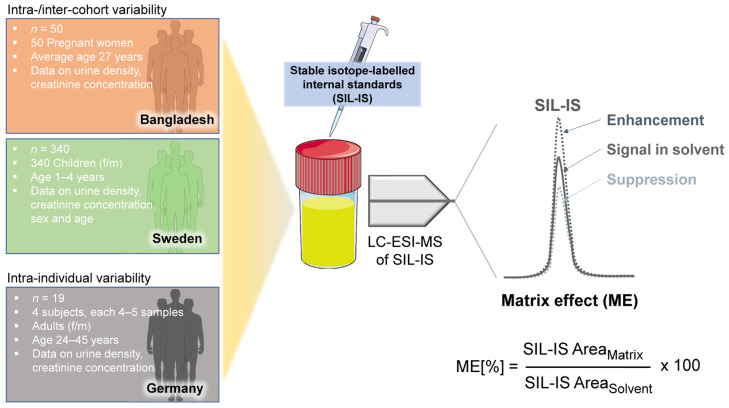
Overview of the experimental approach and involved urine sample cohorts.

The MEs in the LC-ESI-MS analysis of 11 mycotoxins (chemical structures, see Figure S1, Supplementary Materials) were evaluated in urine samples from multiple cohorts to investigate the ME variability within and between cohorts. An overview of the study design and urine cohorts is presented in Figure 1. Stable isotope-labelled internal standards (SIL-IS) were added to all urine samples to determine the ME by comparing the peak areas of the SIL-IS in matrix versus solvent (see Section 5 for details). Limits of detection (LOD), limits of quantification (LOQ), and spiking concentrations are summarized in Table S1 (Supplementary Materials). For a clearer presentation of the results, the MEs obtained for SIL-IS are presented using their unlabelled analytes. Additional subject and sample characteristics, such as sex, age, creatinine concentration, and urine density, were incorporated to support the interpretation of ME variability (Figure 1).

### 2.1. Matrix Effect Variability Within and Between Cohorts

The first cohort comprised 50 urine samples of pregnant Bangladeshi women (average age 27 years), while the second cohort consisted of 340 Swedish children of both sexes, ranging from one to four years. Measured retention times (RTs), including RT range, and percentage of samples with the SIL-IS signal below LOD are summarized in Table S1 (Supplementary Materials). For the Swedish cohort, the proportions of samples without detectable SIL-IS signals were as follows: sterigmatocystin (STG) 3.5%, ochratoxin A (OTA) 0.3%, citrinin (CIT) 2.4%, deoxynivalenol (DON) 1.8%, T-2 toxin (T-2) 0.3%, and HT-2 toxin (HT-2) 7.1%. In the Bangladeshi cohort, only fumonisin B_1_ (FB_1_) and HT-2 showed SIL-IS signals below the LOD, in 2.0% and 56% of samples, respectively.

**Figure 2:**
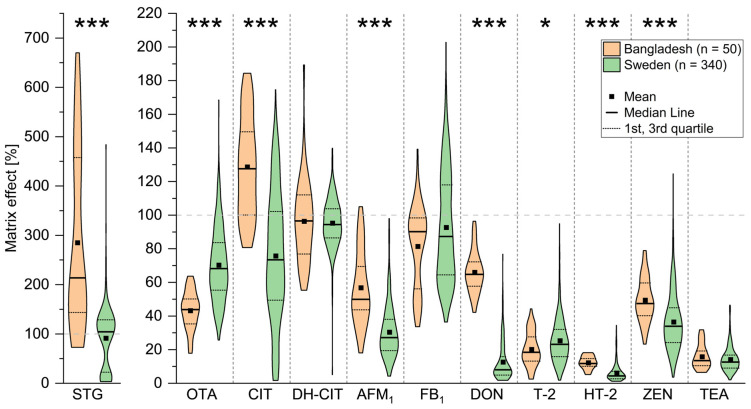
Comparison of matrix effects (ME) within and between cohorts. ME determined for sterigmatocystin (STG), ochratoxin A (OTA), citrinin (CIT), dihydrocitrinone (DH-CIT), aflatoxin M_1_ (AFM_1_), fumonisin B_1_ (FB_1_), deoxynivalenol (DON), T-2 toxin (T-2), HT-2 toxin (HT-2), zearalenone (ZEN), and tenuazonic acid (TEA) in urine samples from the Bangladeshi and Swedish cohorts. The ME was determined using stable isotope-labelled internal standards, added to each urine sample, followed by LC-MS/MS analysis of the spiked standards. Violin plots depict the ME distribution within each cohort. Asterisks indicate significantly different median values between both cohorts determined by the Mann–Whitney test (* 0.05 > *p* > 0.01, *** *p* < 0.001).

The MEs in both cohorts and their variability are compared for each mycotoxin in Figure 2. Details on all descriptive statistics are summarized in Table S4 (Supplementary Materials). Almost all analytes showed signal suppression (ME < 100%), being strongest for HT-2 with a median ME of 12% in the Bangladeshi cohort and 4% in the Swedish cohort. Interestingly, STG, OTA, CIT, dihydrocitrinone (DH-CIT), and FB_1_ showed both signal-suppressing and enhancing effects, especially within the Swedish cohort. The strongest signal enhancement, with a median of 212%, was observed for STG within the Bangladeshi cohort. However, in the Swedish cohort, this was not the case, with a significantly lower median ME of 91% (*p* < 0.001), although single samples also showed strong enhancement of up to 483%. Similarly, less signal suppression or even a reversal to signal enhancement in the Bangladeshi cohort compared to the Swedish cohort was observed for CIT (*p* < 0.001), aflatoxin M_1_ (AFM_1_) (*p* < 0.001), DON (*p* < 0.001), HT-2 (*p* < 0.001), and zearalenone (ZEN) (*p* < 0.001). In contrast, OTA signal suppression was significantly (*p* < 0.001) stronger in the Bangladeshi cohort, with a median ME of 44% compared to 68% in the Swedish cohort. The variability within the cohorts was very high for most mycotoxins. The highest range was observed for STG with 598% in the Bangladeshi cohort and 481% in the Swedish cohort. Equally, the respective interquartile range (IQR, difference between the first and third quartiles) was much higher for the Bangladeshi cohort with 318% than for the Swedish cohort with 107%. All other analytes had higher ranges in the Swedish cohort, e.g., OTA with 46% in the Bangladeshi cohort compared to 143% in the Swedish cohort. There was less variability in ME for mycotoxins with strong signal suppression, such as HT-2 and tenuazonic acid (TEA), with IQRs of 4–9%. Looking into the distribution of the ME, STG showed a bimodal distribution in the Swedish cohort, with a minor mode at strong signal suppression of around 15% and a major mode at around 120%.

### 2.2. Matrix Effect Variability in Individuals

**Figure 3:**
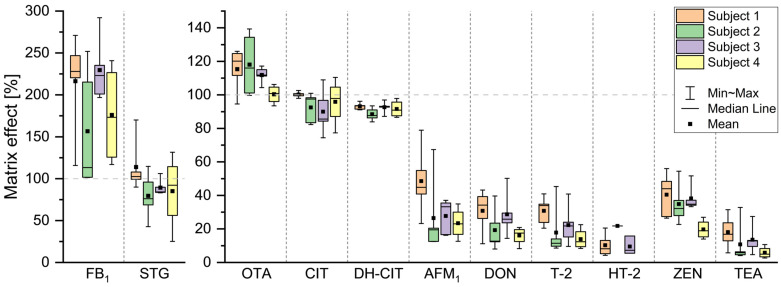
Comparison of inter- and intra-individual variability of matrix effects (ME). ME determined for sterigmatocystin (STG), ochratoxin A (OTA), citrinin (CIT), dihydrocitrinone (DH-CIT), aflatoxin M_1_ (AFM_1_), fumonisin B_1_ (FB_1_), deoxynivalenol (DON), T-2 toxin (T-2), HT-2 toxin (HT-2), zearalenone (ZEN), and tenuazonic acid (TEA) in urine samples of four subjects, each provided 4–5 urine samples within 24 h. The ME was determined using stable isotope-labelled internal standards, added to each sample, followed by LC-MS/MS analysis of the spiked standards. The ME distributions of each subject are shown as box plots with whiskers extending to the minimum and maximum values.

To further break down the variability in MEs, four German volunteers each collected 4–5 urine samples over 24 h, and MEs were determined in each sample. This way, it was possible to determine the extent to which differences within samples from a single individual contribute to overall ME variability, compared with those in urine samples from different individuals. For each subject, ME variabilities are shown as box plots with minimum and maximum whiskers (Figure 3). Most analytes showed mainly signal suppression. OTA showed no signal suppression or even weak signal enhancement, while FB_1_ experienced signal enhancement in all individual samples. Inter-individual differences were again observed. Urine samples from subject 4, on average, showed no signal enhancement for OTA (100% ME), which differed from the slight signal enhancement of about 115% observed in the other subjects. A similar trend was observed for ZEN: urine samples from subject 4 showed an average ME of 20%, while those from subjects 1–3 exerted an average ME of about 38%. For both OTA and ZEN, intra-individual variability ranged from 13% to 40% for OTA and from 13% to 32% for ZEN. Furthermore, the ranges of average ME between subjects for CIT, AFM_1_, DON, T-2, and TEA were 10%, 25%, 15%, 17%, and 12%, while their respective averaged intra-individual ranges were 23%, 38%, 28%, 26%, and 21%. The highest inter- and intra-individual variability was determined for FB_1_. The average ME range across subjects was 73%, while the intra-individual ME range was 95% for subject 3 and up to 155% for subject 1. DH-CIT showed the lowest overall range, with an average of 9% across all subjects. At the same time, the respective inter-individual range of average ME was lowest with 5%.

### 2.3. Factors Influencing Matrix Effects

#### 2.3.1. Sex and Age (Swedish Cohort)

Anonymized sex- and age-related data from the Swedish cohort were analyzed in relation to observed matrix effects for each mycotoxin (Figures S2 and S3, Supplementary Materials). The cohort consisted of 169 boys and 171 girls. For most analytes, the ME distributions were comparable between sexes. Only for AFM_1_ (0.05 > *p* >0.01), DON (0.01 > *p* > 0.001), and HT-2 (0.05 > *p* > 0.01) were statistically significant differences in ME between boys and girls observed (Figure S2, Supplementary Materials). Age distribution was split into children from 1.5 to 2 years (*n* = 64) and from 4 to 4.7 years (*n* = 276). Significant differences in ME between these age groups were detected for STG (0.05 > *p* > 0.01), OTA (*p* < 0.001), CIT (*p* < 0.001), DH-CIT (*p* < 0.001), AFM_1_ (*p* < 0.001), DON (*p* < 0.001), and HT-2 (*p* < 0.001). Notably, for STG, strong signal suppression (ME < 50%) was more frequent in the older age group than in the younger group (Figure S3, Supplementary Materials).

#### 2.3.2. Urine Density and Creatinine Concentration

**Figure 4:**
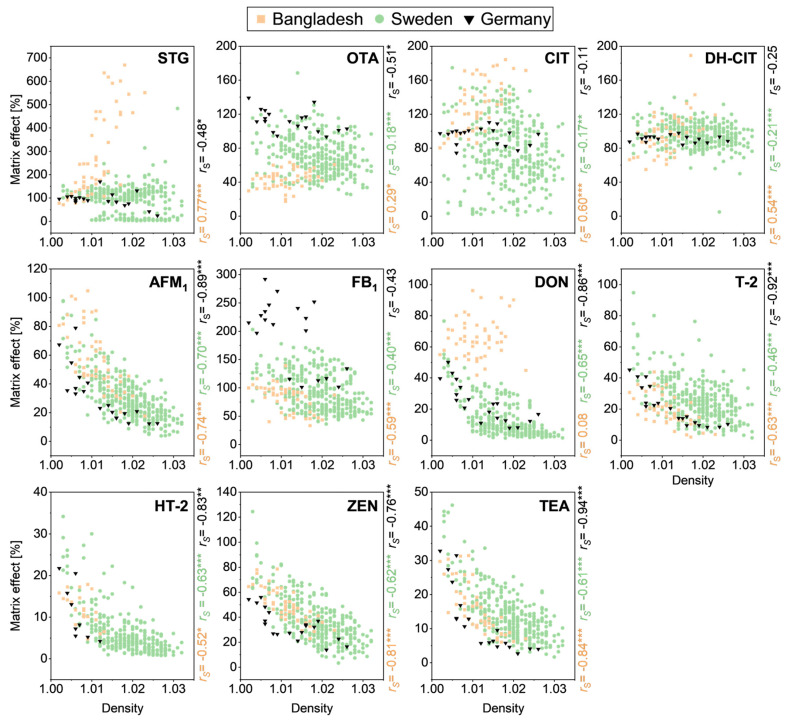
Correlation of matrix effect and urine density. Scatter plots for sterigmatocystin (STG), ochratoxin A (OTA), citrinin (CIT), dihydrocitrinone (DH-CIT), aflatoxin M_1_ (AFM_1_), fumonisin B_1_ (FB_1_), deoxynivalenol (DON), T-2 toxin (T-2), HT-2 toxin (HT-2), zearalenone (ZEN), and tenuazonic acid (TEA) in urine samples from the Bangladeshi (*n* = 50), Swedish (*n* = 340), and German (*n* = 19, from 4 subjects) cohorts. Strength of correlation is indicated by the Spearman correlation coefficient *r*_S_. Asterisks indicate significance of the correlation coefficient (* 0.05 > *p* > 0.01, ** 0.01 > *p* > 0.001, *** *p* < 0.001).

We further investigated the relationship between ME and urine density and creatinine concentration across all three cohorts. Both parameters are used to normalize biomarker concentrations, as they are considered measures of urine concentration that vary based on the hydration status of the subject [17]. Consequently, density and creatinine concentration can be related to the concentration of matrix components that may be responsible for ME. Density and creatinine values differed between cohorts (Table 1). Median density of 1.011 in the Bangladeshi cohort was lower compared to 1.020 in the Swedish cohort and 1.012 in the German cohort. Equally, the first density quartile was 1.008 in the Bangladeshi cohort, 1.014 in the Swedish cohort, and 1.006 in the German cohort. Median creatinine concentrations were 53 mg/100 mL in the Bangladeshi cohort, 68 mg/100 mL in the Swedish cohort, and 73 mg/100 mL in the German cohort. Additionally, the German cohort showed a high IQR, with the first quartile at 21 mg creatinine/100 mL and the third at 142 mg creatinine/100 mL. In the Bangladeshi, Swedish, and German cohorts, urine density and creatinine concentration were strongly correlated, with Spearman’s correlation coefficients (*r*_S_) of 0.85 (*p* < 0.001), 0.87 (*p* < 0.001), and 0.96 (*p* < 0.001), respectively (Table S6, Supplementary Materials). Thus, Figure 4 below presents the respective ME plotted against urine density, while scatter plots of the corresponding data based on creatinine concentration are provided in Figure S4 (Supplementary Materials).

STG showed a strong, positive correlation with density (*r*_S_ = 0.77, *p* < 0.001) in the Bangladeshi cohort. In contrast, the Swedish and German cohorts (*r*_S_ = −0.48, 0.05 > *p* > 0.01) showed no clear correlation. Furthermore, the bimodal distribution of the ME for STG in the Swedish cohort became clearly visible. A subgroup of about 90 samples showed very strong signal suppression well below 50% and distributed across most of the density and creatinine range. The larger subset ranged around 120% ME. This bimodality made the regression not applicable, so no correlation coefficient was calculated.

OTA, CIT, and DH-CIT showed overall weak to moderate correlation with density in both the Swedish (*r*_S_ = −0.18, *p* < 0.001; *r*_S_ = −0.17, 0.01 > *p* > 0.001 and *r*_S_ = −0.21, *p* < 0.001) and German (*r*_S_ = −0.51, 0.05 > *p* > 0.01; *r*_S_ = −0.11, *p* > 0.05 and *r*_S_ = −0.25, *p* > 0.05) cohorts. In contrast, the Bangladeshi cohort again differed from the other two, showing a stronger, positive correlation for CIT (*r*_S_ = 0.60, *p* < 0.001) and DH-CIT (*r*_S_ = 0.54, *p* < 0.001). For DON, however, no correlation with urine density was observed in the Bangladeshi cohort (*r*_S_ = 0.08, *p* > 0.05), whereas the Swedish (*r*_S_ = −0.65, *p* < 0.001) and German (*r*_S_ = −0.86, *p* < 0.001) cohorts showed strong negative correlations, indicating a logarithmic decline of ME concentrations with increasing density.

A strong negative correlation between ME and urine density was observed for AFM_1_, ZEN, and TEA across all cohorts. The corresponding scatter plots display similar patterns among cohorts, although the larger Swedish cohort exhibited greater variability in ME values. In the German cohort, AFM_1_, T-2, and TEA showed a clear logarithmic decline with increasing density, yielding correlation coefficients of *r*_S_ = −0.89, −0.92, and −0.94, respectively (all *p* < 0.001). In contrast, FB_1_ demonstrated only a moderate correlation with density in all cohorts.

#### 2.3.3. Retention Time

Shifts in the RT of an analyte can change the co-eluting matrix environment and thereby possibly influence the ME [7,8]. For most mycotoxins, the RT range across all cohorts (Bangladesh, Sweden, Germany) was minimal (<0.2 min; Table S1, Supplementary Materials). Only CIT in the Swedish cohort, DON in the Swedish and German cohorts, and DH-CIT in all cohorts showed greater variations from 0.265 min to 0.836 min relative to the mean RTs. Among these, DH-CIT in the Bangladeshi cohort and DON in the Swedish and German cohorts showed notable correlations with urine density or creatinine concentration. Specifically, RT of DH-CIT in the Bangladeshi cohort correlated strongly negatively with density (*r*_S_ = −0.87, *p* < 0.001; Table S5, Supplementary Materials). Similarly, RT of DON negatively correlated with density in both the Swedish (*r*_S_ = −0.51, *p* < 0.001) and German (*r*_S_ = −0.84, *p* < 0.001) cohorts. Therefore, partial correlation coefficients *r*_Partial_ were calculated for ME versus RT, with density as the controlling variable (Table S5, Supplementary Materials). Moderate correlations between ME and RT were observed for DH-CIT in the Bangladeshi cohort (*r*_Partial_ = −0.48, *p* < 0.001). In contrast, DON showed no correlation in the Swedish cohort (*r*_Partial_ = 0.05, *p* > 0.05) and only a moderate correlation in the German cohort (*r*_Partial_ = 0.46, *p* > 0.05).

## 3. Discussion

### 3.1. Matrix Effects Greatly Vary and Affect Quantification of Urinary Biomarkers

Comparison of the peak areas of stable isotope-labelled internal standards (SIL-IS) added to urine samples prior to analysis by LC-MS/MS allowed the assessment of matrix effects (MEs). By comparing data from distinct populations in Bangladesh and Sweden, as well as multiple samples from a small cohort in Germany, different correlation analyses were made to identify factors related to MEs. Generally, a broad range of median MEs from 4% to 214% was observed across all analytes and cohorts, and high variability was observed both between and within the cohorts. These findings are in good agreement with those of a study on the MEs of 51 different drugs in 338 urine samples. In this study, the range of MEs across all analytes was from complete suppression to up to almost 200% signal enhancement. The averaged intra-analyte difference in MEs between samples was calculated at 156% [18]. Other studies with fewer urine samples reported smaller ME ranges [7,19]. In general, the sample preparation strategy influences the magnitude of MEs, with highly selective clean-up methods, such as immunoaffinity columns, achieving efficient matrix removal and thus less pronounced ME [20,21]. It can be expected that extensive sample clean-up also reduces ME variability. However, human biomonitoring studies require large sample numbers, which puts laborious clean-up strategies at a disadvantage. Therefore, recent methods for mycotoxin biomonitoring in urine still rely on unspecific, low-effort sample preparation strategies, accepting partly strong MEs [22,23].

The guideline on “Bioanalytical Method Validation and Study Sample Analysis”, harmonized by the European Medicines Agency and the American Food and Drug Administration, allows a coefficient of variation of 15% for the ME, determined across six different matrix sources [10]. In our much larger cohorts, this reference value was only fulfilled for DH-CIT in the Swedish cohort. The Swedish cohort had the highest overall ME variability based on the range of observed ME. However, the sample size was almost seven times that of the Bangladeshi cohort, naturally leading to greater variability. For the study purpose, we used the analyte SIL-IS to assess the ME, enabling ME compensation for quantification. However, suitable SIL-IS are not always available or are very costly. In those cases, the high variability of ME can lead to incorrect results when matrix(-matched) calibration is used to quantify mycotoxins in urine samples. Assuming the blank urine used for calibration causes a ME at the median level of each mycotoxin in the respective cohort, the relative error for samples with weaker or stronger ME can be calculated. For STG, this would result in overestimates of 215% and 124% at the 75th percentile level for the Bangladeshi and Swedish cohorts, respectively. At the 25th percentile, an underestimate to 66% or even 21% of the real concentration would result. In the Bangladeshi cohort, calculated concentrations of OTA, CIT, DH-CIT, and DON are within acceptable ranges of >70% and <120%, whereas in the Swedish cohort, this is achieved only for DH-CIT. This effect can be even more pronounced when the used blank urine is from a very different population. Substantial variations in ME must be taken into account when using human biomonitoring data from urine sample analysis for exposure assessment.

### 3.2. Matrix Effects Influence Sensitivity and Uncertainty of Exposure Calculations

Regarding method performance, another aspect affected by the ME is sensitivity. For some analytes, the ME distribution is skewed to the right. Initially, it could be assumed that strong signal suppression led to undetectable signals (i.e., <LOD or signal-to-noise ratio <3), resulting in left-censored data. However, this can only be assumed relevant for HT-2, for which 56% of the Bangladeshi samples and 7% of the Swedish samples were below the LOD. Given the strong signal suppression and the SIL-IS spiking concentration 24 times above the LOD, the HT-2 signal is more likely to fall below the LOD than the other analytes, which were spiked at concentrations at least 30 times above the LOD and experienced less signal suppression. The lower spiking level above LOD was chosen to reduce SIL-IS consumption given the relatively high LOD of HT-2 (0.833 ng/mL). For HT-2, the different percentages of samples <LOD between the Bangladeshi and Swedish cohorts did not correspond to the different MEs. Nevertheless, for STG, CIT, and DON, the stronger signal suppression in the Swedish cohort corresponded to a higher percentage of not detectable SIL-IS signals compared to the Bangladeshi cohort. This shows how the method’s sensitivity is dependent on the analyzed cohort but even more on the individual urine sample. Consequently, careful monitoring of the actual sensitivity is necessary, especially when exposure calculations include the LODs and LOQs of biomarkers [24].

### 3.3. Matrix Effects May Vary Depending on Diet

Considering the influence of factors such as origin, diet, age, sex, and other determinants of urine composition, it is not surprising that the median MEs of STG, OTA, CIT, AFM_1_, DON, T-2, HT-2, and ZEN differed significantly between the Bangladeshi and Swedish cohorts. Although it has been shown that the sex of subjects has an influence on urine characteristics like pH [14], levels of endogenous metabolites (e.g., lactate, dimethylamine, citrate) [15], and overall urine concentration [16], little to no influence on ME was observed in the Swedish cohort. This may be attributed to the relatively young age of the study participants. In contrast, the two age groups in the Swedish cohort differed more in their ME distribution. Especially for OTA, CIT, and DON, the median values vary significantly between the two groups. The influence of age on human urine components has also been reported for various excretory products, including glycine, creatinine, and carnitine [11,15]. In this case, the age difference in both groups is also associated with a changing, more diverse diet as the children grow older. The human diet is reflected in the urine composition [12,13], and in fact, urinary biomarkers of food intake are used to evaluate individual nutrition [25]. Therefore, differences in ME between the two age groups and between the Swedish and Bangladeshi cohorts might be due to differences in dietary habits.

### 3.4. The Variability in Matrix Effects Is Correlated with Urine Concentration for Some Analytes

Yet, these factors do not explain the high intra-individual variability observed for four German subjects. Variability within subjects actually exceeded the variability between subjects for all mycotoxins. The results suggest that, within a relatively homogeneous cohort, variability in ME is primarily attributed to intra-individual fluctuations in urine composition over 24 h rather than to inter-individual differences. However, the small size of that cohort limits the significance of the results and further confirmation with more samples and different populations is required. Nevertheless, this observation is supported by the results on the correlation of ME with density and creatinine, both parameters reflecting urine concentration.

Notably, the more polar analytes AFM_1_, DON, T-2, and TEA, as well as the less polar ZEN, showed strong negative correlations between ME and urine density or creatinine concentration, indicating a stronger ME with increasing concentration. In contrast, differences between the Bangladeshi and the two European cohorts for STG, CIT, DH-CIT, and DON likely reflect cohort-specific factors discussed earlier. Polar analytes are known to be more prone to signal suppression [6,26], which is in agreement with our results. The ME of the less polar OTA, CIT, and DH-CIT only correlated weakly with density and creatinine. At the same time, these analytes experienced rather moderate signal suppression or even an enhanced signal by the urine matrix. Stahnke et al. [9] and Kruve et al. [27] performed dilution experiments of pesticides in food matrices to investigate the behaviour of ME with sample dilution. Interestingly, their dilution graphs have strong similarities with our plots of ME and urine density/creatinine concentration. Their studies show that for analytes with very strong signal suppression, the plots of ME versus dilution factor follow a logarithmic curve that levels off close to the undiluted sample. A comparable logarithmic trend was observed here for DON, T-2, HT-2, and TEA, all of which showed strong signal suppression, when ME was plotted against urine density or creatinine concentration. In their experiments, the curve flattened at high dilutions, where ME became negligible; this was not observed in our data, likely because no additional sample dilution was performed.

For ZEN, showing overall less signal suppression, a more linear trend was observed between ME and density, while the plots against creatinine concentration still followed a logarithmic curve. A connection between ME and urine creatinine concentration was also observed in another study, showing that urine samples with low creatinine concentration are associated with less signal suppression for some analytes [7]. It can be assumed that, for the analytes demonstrating a consistent correlation of ME with urine density or creatinine concentration in all cohorts, i.e., AFM_1_, T-2, HT-2, ZEN, and TEA, the ME is independent of the individual characteristics of the subjects and might be attributed to constitutively excreted, endogenous compounds. Therefore, the differences observed in median ME between the Bangladeshi and Swedish cohorts are likely attributable to differences in the distributions of urine density and creatinine concentrations rather than to intrinsic cohort-specific factors.

### 3.5. Retention Time Shifts May Also Lead to Variability of Matrix Effects

Sample matrix load can have an impact on the chromatographic separation of analytes and mostly lead to slightly shorter retention times. Indeed, partial correlation regression revealed a moderate impact of RT on the ME of DH-CIT in the Bangladeshi cohort and of DON in the German cohort. The respective RTs correlated negatively with the density/creatinine values, as the active sites of the stationary phase were being occupied by matrix components. Findings on the ME being dependent on the RT have already been described [8,28], corresponding with our observations.

## 4. Conclusions

Understanding and overcoming MEs in LC-MS is still an ongoing issue. In particular, the analysis of biological samples like urine poses a difficult task in analytical method development and validation. Using stable isotope-labelled standards, we investigated the variability of ME within and between different urine sample cohorts. The significant differences observed between cohorts, but also their respective high variability, which affects method sensitivity, an important parameter for trace analysis of, e.g., biomarkers of mycotoxin exposure. Our findings highlight that quantification solely by matrix(-matched) calibration using blank urine must be carefully applied. Ideally, a blank matrix from the cohort to be analyzed should be used, avoiding biases by additional population-dependent ME. Nonetheless, we could show that for some analytes, the ME does correlate with urine concentration, as reflected in urine density and creatinine concentration. Accordingly, quantitative results obtained by matrix(-matched) calibration of samples with very high or low urine concentration should be diluted or excluded from analysis for certain analytes. Ultimately, reducing ME to an acceptable level remains the most reliable strategy for robust analysis, particularly when stable isotope-labelled standards are unavailable.

## 5. Materials and Methods

### 5.1. Chemicals and Reagents

Acetonitrile (LC-MS grade) was purchased from Fisher Scientific (Schwerdte, Germany). Ultrapure water ASTM type I (>18 MΩ) was produced with a Purelab Flex system (ELGA, Bielefeld, Germany). Formic acid and creatinine were purchased from Merck KGaA (Darmstadt, Germany). *d*_5_-ochratoxin A (*d*_5_-OTA), ^13^C_3_-citrinin (^13^C_3_-CIT), ^13^C_3_-dihydrocitrinone (^13^C_3_-DH-CIT), ^13^C_2_-tenuazonic acid (^13^C_2_-TEA), *d*_3_-T-2 toxin (*d*_3_-T-2), *d*_9_-HT-2 toxin (*d*_9_-HT-2), *d*_1_-deoxynivalenol (*d*_1_-DON), and *d*_2_-zearalenone (*d*_2_-ZEN) were chemically synthesized [29]. U-[^13^C_17_]-aflatoxin M_1_ (U-[^13^C_17_]-AFM_1_) was purchased from Romer Labs Deutschland GmbH (Butzbach, Germany). U-[^13^C_18_]-sterigmatocystin (U-[^13^C_18_]-STG) was purchased from Fianovis (Vindry-sur-Turdine, France). ^13^C_34_-fumonisin B_1_ (^13^C_34_-FB_1_) was purchased from Sigma-Aldrich (Taufkirchen, Germany). A mix of all SIL-IS was used at a 100-fold concentration of the final concentration needed in the urine sample.

### 5.2. Urine Samples

Variability of ME within and between cohorts was investigated in a cohort of 50 pregnant women from Bangladesh (average age 27 years) drawn from the Maternal Exposure to Mycotoxins and Adverse Pregnancy Outcomes cohort (MEMAPO study) [30], and in a cohort of 340 Swedish children (1.5- and 4-year-olds, female and male) from the Riksmaten Young Children study (Swedish Ethical Review Authority ref no 2020-05293) conducted by the Swedish Food Agency [31]. The samples of the MEMAPO study have already been analyzed for mycotoxins, and the results are published elsewhere [32]. After sampling, all samples were immediately (within the same day) stored at −80 °C until further analysis. ME variability in individual subjects was further assessed in four German volunteers (one female, three males, ages 24–45), each of whom provided four to five urine samples over a 24 h period. Ethical approval was obtained, and all volunteers gave consent to anonymized sample processing [25]. Prior to urine collection, all volunteers abstained from cereals and cereal-based foods for approximately 36 h. After collection, the samples were stored at −20 °C until further analysis.

Urine density and creatinine concentration of the Bangladeshi cohort samples were determined at the central laboratory of Heidelberg University Hospital [32]. For urine density, multi-parameter test strips on a CLINITEK Novus^®^ analyzer (Siemens Healthcare GmbH, Erlangen, Germany) were used. Creatinine concentrations were quantified on a ADVIA XPT chemistry analyzer (Siemens Healthineers AG, Forchheim, Germany). For the Swedish cohort, urinary creatinine was determined by a fully automated direct enzymatic method using Abbott Architect ci8200 analyzer (Abbott Laboratories, Abbott Park, IL, USA) at the Finnish Institute for Health and Welfare (THL), Helsinki, Finland. Specific gravity (converted to density assuming a water density of 1.0 g/cm^3^) was determined using a digital refractometer (Refractometer 30PX, Mettler Toledo, Columbus, OH, USA) at the Division of Occupational and Environmental Medicine, Lund University, Sweden. For the German cohort, urine density was determined on an EasyDens instrument (Anton Paar Germany GmbH, Ostfildern-Scharnhausen, Germany). Determination of the creatinine concentration was done using a modified Jaffe method [33,34]. Up to three freeze–thaw cycles were required for aliquotation and analysis of density, creatinine, mycotoxins and metabolites.

### 5.3. Sample Preparation and Spiking

Urine samples were thawed at room temperature and vortexed, and 297 µL were transferred into a 96-well plate with a conical bottom, followed by the addition of 3 µL of the SIL-IS mix (Table S1, Supplementary Materials). The SIL-IS mix was also added in the same concentration to an eight-point calibration curve in water. Details on the calibration curves are listed in Table S2 (Supplementary Materials). The mean peak area of each SIL-IS in the calibration, as well as the SIL-IS peak area in the spiked urine samples, was later used for ME calculations. Final spiked concentration of each SIL-IS is summarized in Table S1 (Supplementary Materials). The spiking concentrations were chosen based on the centre of the calibration range or slightly above for each mycotoxin. The whole plate was then put on an orbital shaker (Matrix Orbital Delta Plus, IKA GmbH, Staufen, Germany) for 10 min at 550 rpm, followed by centrifugation for 10 min at 3200× *g*. The sample plate was then subjected to online solid-phase extraction-LC-MS/MS analysis.

### 5.4. Online Solid-Phase Extraction-LC-MS/MS Method

Analysis of urine samples was performed using a recently published online solid-phase extraction (SPE)-LC-MS/MS method [29]. Briefly, the setup consisted of two quaternary pumps (1260 Infinity II Bio-inert LC system; Agilent, Waldbronn, Germany), one for the SPE column (Oasis HLB; 2.1 × 20 mm, 5 µm; Waters GmbH, Eschborn, Germany) and one for the analytical column (Nucleodur Phenyl-Hexyl; 2 × 100 mm, 3 µm; Macherey-Nagel, Düren, Germany) coupled to a Sciex 7500 QTRAP-activated mass spectrometer (Sciex, Darmstadt, Germany). Both pumps used water and acetonitrile with formic acid added. A 100 µL measure of undiluted urine was used for injection. The samples were cleaned and purified in multiple steps on the online SPE before being transferred to the analytical column. Flow dilution was used to reduce the organic content of the SPE eluate and refocus the analytes, followed by gradient elution. Analytes were ionized by electrospray ionization at both polarities in a single run. Spray voltages were set to ±3000 V at a source temperature of 450 °C. Nebulizer gas (gas 1), drying gas (gas 2), and curtain gas were set to 65 psi, 50 psi, and 50 psi, respectively. Scheduled multiple reaction monitoring was applied with a target cycle time of 400 ms and a RT window of 30 to 60 s. For each compound, two to three transitions were recorded for reliable qualification. STG, OTA, CIT, DH-CIT, AFM_1_, FB_1_, DON, T-2, and HT-2 were ionized in positive polarity. ZEN and TEA were ionized in negative polarity. Further information on multiple reaction monitoring transitions and MS parameters used are listed in Table S3 (Supplementary Materials). Full details on the online SPE-LC-MS/MS method are described in Kuhn et al. [29].

### 5.5. Determination of Matrix Effects

The absolute ME was determined as described by Matuszewski et al. [3]. Because the urine samples were naturally contaminated with mycotoxins, spiking SIL-IS into the samples and a solvent calibration in water (see Section 5.3) still allowed for the assessment of ME. The peak area ratio of equal amounts of the SIL-IS spiked in urine matrix (SIL-IS Area_Spiked Matrix_) compared to the SIL-IS in pure solvent, averaged across eight calibration points (SIL-IS Area_Solvent_), was used to calculate the ME. Values below 100% indicate signal suppression, while values above 100% indicate signal enhancement.

ME [%] = (SIL-IS Area_Spiked Matrix_/SIL-IS Area_Solvent_) × 100 (1)

### 5.6. Statistical Analysis

Statistical analysis was performed using OriginPro 2025 (Vers. 10.2.0.196). The distributions of ME, urine density, creatinine concentration, and RT were assessed for normality using Shapiro–Wilk’s test. As normality was rejected for most variables, subsequent statistical analyses were performed using methods that account for non-normal distributions. Differences between cohorts and groups were compared using the Mann–Whitney test. Strength of correlations was calculated as the Spearman correlation coefficient *r*_S_ or the partial correlation coefficient *r*_Partial_. Statistical significance was determined at the 95% confidence level.

## Figures and Tables

**Table 1:** Statistics for urine densities and creatinine concentrations in the Bangladeshi, Swedish, and German cohorts.

	Bangladeshi Cohort		Swedish Cohort		German Cohort	
	Density	Creatinine [mg/100 mL]	Density ^a^	Creatinine [mg/100 mL]	Density	Creatinine [mg/100 mL]
Mean	1.011	61	1.019	71	1.012	80
Median	1.011	53	1.020	68	1.012	73
1st quartile	1.008	38	1.014	45	1.006	21
3rd quartile	1.014	77	1.024	90	1.018	142
Min	1.002	12	1.003	5	1.002	14
Max	1.023	176	1.032	207	1.026	212

^a^ Calculated from specific gravity, assuming a water density of 1.0 g/cm^3^.

## Data Availability

The original contributions presented in this study are included in the article/Supplementary Materials. Further inquiries can be directed to the corresponding authors.

## Supplementary Materials

The following supporting information can be downloaded at: https://www.mdpi.com/article/10.3390/toxins18030135/s1, Table S1: Details on the experimental setup and results of analysis; Table S2: Details on the calibration curves; Table S3: Details on mass spectrometric parameters; Table S4: Detailed statistics on the matrix effect distributions in the Bangladeshi (B) and Swedish (S) cohorts; Table S5: Correlations between retention time (RT) and density, as well as matrix effect (ME) and retention time for dihydrocitrinone (DH-CIT) and deoxynivalenol (DON); Table S6: Correlation of urine density and creatinine concentration; Figure S1: Chemical structures of the 11 mycotoxins, for which matrix effects were studied in urine samples; Figure S2: Influence of subjects’ sex on matrix effects; Figure S3: Influence of subjects’ age on matrix effects; Figure S4: Correlation of matrix effect and creatinine concentration.

## Abbreviations

The following abbreviations are used in this manuscript:

ME	Matrix effect
SIL-IS	Stable isotope-labelled standards
IQR	Interquartile range
LC-ESI-MS/MS	Liquid chromatography coupled with electrospray ionization-tandem mass spectrometry
RT	Retention time
ICH	International Council for Harmonisation of Technical Requirements for Pharmaceuticals for Human Use
LOD	Limit of detection
LOQ	Limit of quantification
STG	Sterigmatocystin
OTA	Ochratoxin A
CIT	Citrinin
DON	Deoxynivalenol
T-2	T-2 toxin
HT-2	HT-2 toxin
FB_1_	Fumonisin B_1_
DH-CIT	Dihydrocitrinone
AFM_1_	Aflatoxin M_1_
ZEN	Zearalenone
TEA	Tenuazonic acid
