# Supplementary material for: Matrix Effect Variability in Urine Samples from Different Cohorts and Implications on LC-ESI-MS Mycotoxin Biomarker Analysis
Source: Toxins (Basel). 2026 Mar 10;18(3):135. doi: 10.3390/toxins18030135 (PMC13030103; doi:10.3390/toxins18030135)
Supplement: Supplementary file 1 [file toxins-18-00135-s001.zip › toxins-4137625-supplementary.pdf]

Supplementary materials

Matrix effect variability in urine samples from different cohorts and implications on LC-ESI-MS mycotoxin biomarker analysis

Michael Kuhn[1], Åsa Svanström[2], Nicholas N. A. Kyei[3], Sanna Lignell[2], Hans-Ulrich Humpf[1,*], Benedikt Cramer[1,*]

[1] Institute of Food Chemistry, University of Münster, Corrensstraße 45, 48149 Münster, Germany

[2] Risk Benefit Assessment Department, Swedish Food Agency, PO Box 622, 75126 Uppsala, Sweden

[3] Institute of Public Health, Charité–Universitätsmedizin Berlin, corporate member of Freie Universität Berlin and Humboldt-Universität zu Berlin, Charitéplatz 1, 10117 Berlin, Germany; Research Department 2, Potsdam Institute for Climate Impact Research (PIK), Member of the Leibniz Association, P. O. Box 60 12 03, 14412, Potsdam, Germany; Heidelberg Institute of Global Health, Heidelberg University, Im Neuenheimer Feld 324, 69120 Heidelberg, Germany

* Corresponding authors: Benedikt Cramer, Hans-Ulrich Humpf,

Phone: +49 251 83 33391; Email: cramerb@uni-muenster.de (BC), humpf@uni-muenster.de (HUH)

Table S1 Details on the experimental setup and results of analysis. Limits of detection (LOD) and quantification (LOQ) of the used online SPE-LC-MS/MS method are given with the respective concentrations the stable isotope-labelled internal standards (SIL-IS) are spiked into the samples. For the three cohorts, retention time (RT) with respective range as well as the percentage of samples with the SIL-IS signal below LOD (signal-to-noise <3). Note that RTs are relatively high because of the prior online sample clean-up.

Analyte	LOD / LOQ urine [ng/mL]	Spiked SIL-IS conc [ng/mL]	Bangladeshi cohort		Swedish cohort		German cohort	
			Mean RT (Range) [min]	Samples IS <LOD [%]	Mean RT (Range) [min]	Samples IS <LOD [%]	Mean RT (Range) [min]	Samples IS <LOD [%]
STG	0.001 / 0.002	0.03	20.576 (0.027)	0	20.602 (0.067)	3.5	20.565 (0.008)	0
OTA	0.002 / 0.008	0.11	20.460 (0.023)	0	20.481 (0.055)	0.3	20.457 (0.011)	0
CIT	0.062 / 0.125	2.0	20.068 (0.051)	0	20.083 (0.265)	2.4	20.082 (0.041)	0
DH-CIT	0.003 / 0.016	0.21	22.949 (0.769)	0	22.940 (0.836)	0	23.047 (0.378)	0
AFM_1	0.01 / 0.06	0.80	18.184 (0.021)	0	18.191 (0.041)	0	18.177 (0.014)	0
FB_1	0.001 / 0.005	0.15	17.852 (0.019)	2.0	17.859 (0.087)	0	17.856 (0.013)	0
DON	0.10 / 0.67	4.4	10.781 (0.132)	0	10.794 (0.318)	1.8	10.714 (0.307)	0
T-2	0.020 / 0.080	1.1	19.920 (0.024)	0	19.945 (0.070)	0.3	19.906 (0.007)	0
HT-2	0.833 / 5.0	20	18.988 (0.020)	56	19.008 (0.084)	7.1	18.979 (0.008)	32
ZEN	0.005 / 0.027	0.53	20.303 (0.022)	0	20.329 (0.073)	0	20.290 (0.008)	0
TEA	0.064 / 0.192	5.0	18.622 (0.042)	0	18.655 (0.129)	0	18.622 (0.027)	0

Table S2 Details on calibration curves for each mycotoxin including the concentration (c), average peak area (*n* = 3), and standard deviation (SD) for each calibration point (1–8).

		**1**	**2**	**3**	**4**	**5**	**6**	**7**	**8**
STG	c [ng/mL]	0.002	0.004	0.007	0.013	0.027	0.040	0.093	0.200
	Peak area [counts]	$3.22*10^4$	$5.91*10^4$	$7.73*10^4$	$1.90*10^5$	$4.98*10^5$	$9.23*10^5$	$2.92*10^6$	$9.85*10^6$
	SD [counts]	$4.28*10^3$	$6.83*10^3$	$1.65*10^4$	$1.12*10^4$	$3.57*10^4$	$1.22*10^5$	$2.57*10^5$	$1.09*10^6$
OTA	c [ng/mL]	0.008	0.016	0.027	0.053	0.107	0.160	0.373	0.800
	Peak area [counts]	$1.67*10^5$	$3.95*10^5$	$5.51*10^5$	$1.15*10^6$	$2.39*10^6$	$3.16*10^6$	$7.32*10^6$	$1.74*10^7$
	SD [counts]	$1.11*10^4$	$1.30*10^4$	$4.17*10^4$	$1.05*10^5$	$9.90*10^4$	$4.05*10^4$	$5.79*10^5$	$1.57*10^6$
CIT	c [ng/mL]	0.125	0.250	0.416	0.832	1.66	2.50	5.82	12.5
	Peak area [counts]	$9.51*10^5$	$1.87*10^6$	$2.99*10^6$	$5.48*10^6$	$1.19*10^7$	$1.77*10^7$	$4.11*10^7$	$9.53*10^7$
	SD [counts]	$7.69*10^4$	$2.60*10^5$	$2.01*10^5$	$3.40*10^5$	$9.75*10^5$	$1.36*10^6$	$6.98*10^5$	$5.28*10^6$
DH-CIT	c [ng/mL]	0.016	0.032	0.053	0.11	0.21	0.32	0.75	1.6
	Peak area [counts]	$1.42*10^5$	$2.08*10^5$	$2.83*10^5$	$4.91*10^5$	$9.38*10^5$	$1.39*10^6$	$3.03*10^6$	$6.92*10^6$
	SD [counts]	$1.28*10^4$	$6.98*10^3$	$1.95*10^4$	$3.61*10^4$	$4.40*10^4$	$6.60*10^4$	$1.50*10^5$	$4.80*10^5$
AFM_1	c [ng/mL]	0.06	0.1	0.2	0.4	0.8	1.2	2.8	6
	Peak area [counts]	$7.52*10^5$	$1.44*10^6$	$2.62*10^6$	$4.71*10^6$	$1.07*10^7$	$1.47*10^7$	$3.50*10^7$	$7.29*10^7$
	SD [counts]	$1.26*10^5$	$1.11*10^5$	$4.05*10^5$	$4.90*10^5$	$1.20*10^6$	$7.95*10^5$	$2.88*10^6$	$7.47*10^6$
FB_1	c [ng/mL]	0.005	0.011	0.018	0.035	0.071	0.106	0.247	0.530
	Peak area [counts]	$3.12*10^4$	$5.74*10^4$	$9.32*10^4$	$1.77*10^5$	$3.69*10^5$	$6.26*10^5$	$1.22*10^6$	$2.93*10^6$
	SD [counts]	$3.29*10^3$	$3.75*10^3$	$8.25*10^3$	$1.33*10^4$	$1.97*10^4$	$2.22*10^4$	$5.86*10^3$	$3.15*10^5$
DON	c [ng/mL]	0.67	1.33	2.22	4.44	8.89	13.3	31.1	66.7
	Peak area [counts]	$2.52*10^5$	$4.74*10^5$	$7.72*10^5$	$1.35*10^6$	$3.07*10^6$	$4.05*10^6$	$9.00*10^6$	$2.51*10^7$
	SD [counts]	$8.56*10^3$	$4.94*10^4$	$4.08*10^4$	$1.23*10^5$	$8.01*10^4$	$3.76*10^5$	$7.89*10^5$	$8.14*10^5$
T-2	c [ng/mL]	0.08	0.16	0.27	0.53	1.07	1.60	3.73	8.00
	Peak area [counts]	$1.92*10^5$	$3.54*10^5$	$4.68*10^5$	$1.02*10^6$	$2.87*10^6$	$4.76*10^6$	$1.11*10^7$	$3.32*10^7$
	SD [counts]	$4.82*10^4$	$2.87*10^4$	$9.47*10^4$	$2.15*10^5$	$5.57*10^5$	$5.00*10^5$	$2.38*10^6$	$2.79*10^6$
HT-2	c [ng/mL]	1	2	3	7	13	20	47	100
	Peak area [counts]	$1.11*10^5$	$2.71*10^5$	$4.07*10^5$	$5.57*10^5$	$1.39*10^6$	$2.08*10^6$	$4.53*10^6$	$1.21*10^7$
	SD [counts]	$9.65*10^3$	$3.76*10^4$	$6.89*10^4$	$6.43*10^4$	$8.62*10^4$	$3.07*10^5$	$5.90*10^5$	$1.16*10^6$
ZEN	c [ng/mL]	0.03	0.05	0.09	0.18	0.36	0.53	1.24	2.67
	Peak area [counts]	$4.66*10^4$	$1.06*10^5$	$1.37*10^5$	$3.26*10^5$	$8.46*10^5$	$1.37*10^6$	$3.82*10^6$	$1.25*10^7$
	SD [counts]	$9.99*10^3$	$4.04*10^3$	$2.45*10^4$	$2.15*10^4$	$8.14*10^4$	$8.39*10^4$	$1.78*10^5$	$1.07*10^6$
TEA	c [ng/mL]	0.192	0.384	0.640	1.28	2.56	3.84	8.96	19.2
	Peak area [counts]	$1.90*10^5$	$3.73*10^5$	$5.84*10^5$	$1.14*10^6$	$2.61*10^6$	$3.86*10^6$	$8.47*10^6$	$2.23*10^7$
	SD [counts]	$9.90*10^3$	$4.98*10^4$	$2.96*10^4$	$1.80*10^4$	$1.32*10^5$	$1.95*10^5$	$1.24*10^5$	$8.87*10^5$

Table S3 Details on scheduled MRM transitions and MS parameters including entrance potential (EP), collision energy (CE), and collision cell exit potential (CXP).

Analyte	Q1 mass [*m/z*]	Q3 mass [*m/z*]	RT Window [sec]	EP [V]	CE [V]	CXP [V]
$^{13}C_{17}$-AFM$_1$	346.1	288.1	30	10	36	15
	346.1	317.1		10	28	15
$^{13}C_{18}$-STG	343.1	297.1	30	6	53	45
	343.1	197.1		6	15	45
	343.1	327.1		6	37	45
$^{13}C_{34}$-FB$_1$	756.5	356.4	30	10	55	15
	756.5	374.4		10	50	15
$^{13}C_3$-CIT	254.1	94.0	30	10	56	13
	254.1	118.0		10	68	13
	254.1	236.0		10	23	22
$^{13}C_3$-DH-CIT	270.1	206.1	60	10	40	14
	270.1	234.0		10	35	19
d_5-OTA	409.1	239.0	30	10	36	14
	409.1	221.0		10	50	16
d_2-ZEN	319.2	177.0	30	-10	-34	-12
	319.2	133.0		-10	-40	-12
d_3-T-2	492.2 [M+Na]+	390.2	30	10	29	13
	492.2 [M+Na]+	330.2		10	34	13
d_9-HT-2	456.2 [M+Na]+	345.0	30	10	25	11
	456.2 [M+Na]+	285.0		10	30	11
d_1-DON	298.1	231.0	30	10	17	11
	298.1	219.0		10	17	11
$^{13}C_2$-TEA	198.1	141.0	30	-10	-28	-15
	198.1	114.0		-10	-32	-15

Table S4 Detailed statistics on the matrix effect distributions in the Bangladeshi (B) and Swedish (S) cohorts.

Parameter/	Mean		Median		Min		Max		Range		IQR[a]		1st quartile		3rd quartile	
Analyte	B	S	B	S	B	S	B	S	B	S	B	S	B	S	B	S
STG	285	91	214	104	72	2	670	483	598	481	318	107	142	22	460	129
OTA	43	70	44	68	17	25	63	168	46	143	15	28	35	55	50	84
CIT	129	76	128	73	80	1	184	174	104	173	51	53	100	49	151	102
DH-CIT	96	95	97	94	55	5	189	140	134	135	36	17	77	86	112	104
AFM_1	57	30	50	27	18	4	105	98	87	94	27	19	43	19	70	38
FB_1	81	93	90	87	33	36	139	203	106	167	43	54	56	64	98	118
DON	66	13	65	8	42	1	96	76	54	75	15	11	57	5	72	16
T-2	20	25	18	23	2	1	44	95	42	93	15	16	13	16	28	32
HT-2	12	6	12	4	5	1	18	34	13	33	5	4	10	3	15	7
ZEN	49	36	47	34	23	3	79	124	56	121	20	21	40	24	60	45
TEA	16	14	13	13	6	2	31	46	25	45	9	8	10	9	19	17

[a] Interquartile range

Table S5 Correlations between retention time (RT) and density, as well as matrix effect (ME) and retention time for dihydrocitrinone (DH-CIT) and deoxynivalenol (DON). The correlation coefficients regarding RT *vs* density were calculated by Spearman coefficient r_S, and ME *vs* RT were calculated by partial correlation coefficient $r_{Partial}$, setting density as the controlling variable. Asterisks indicate significance of the correlation coefficient (*** p <0.001).

	**Bangladeshi cohort**		**Swedish cohort**		**German cohort**	
	r_S	$r_{Partial}$	r_S	$r_{Partial}$	r_S	$r_{Partial}$
DH-CIT	-0.87***	-0.48***		-		-
DON	-	-	-0.51***	0.05	-0.84***	0.46

Table S6 Correlations between density and creatinine concentration in the Bangladeshi, Swedish, and German cohorts. Strength of correlation is indicated by the Spearman correlation coefficient r_S. Asterisks indicate significance of the correlation coefficient (*** $p < 0.001$).

	Bangladeshi cohort	Swedish cohort	German cohort
r_S	0.85***	0.87***	0.96***

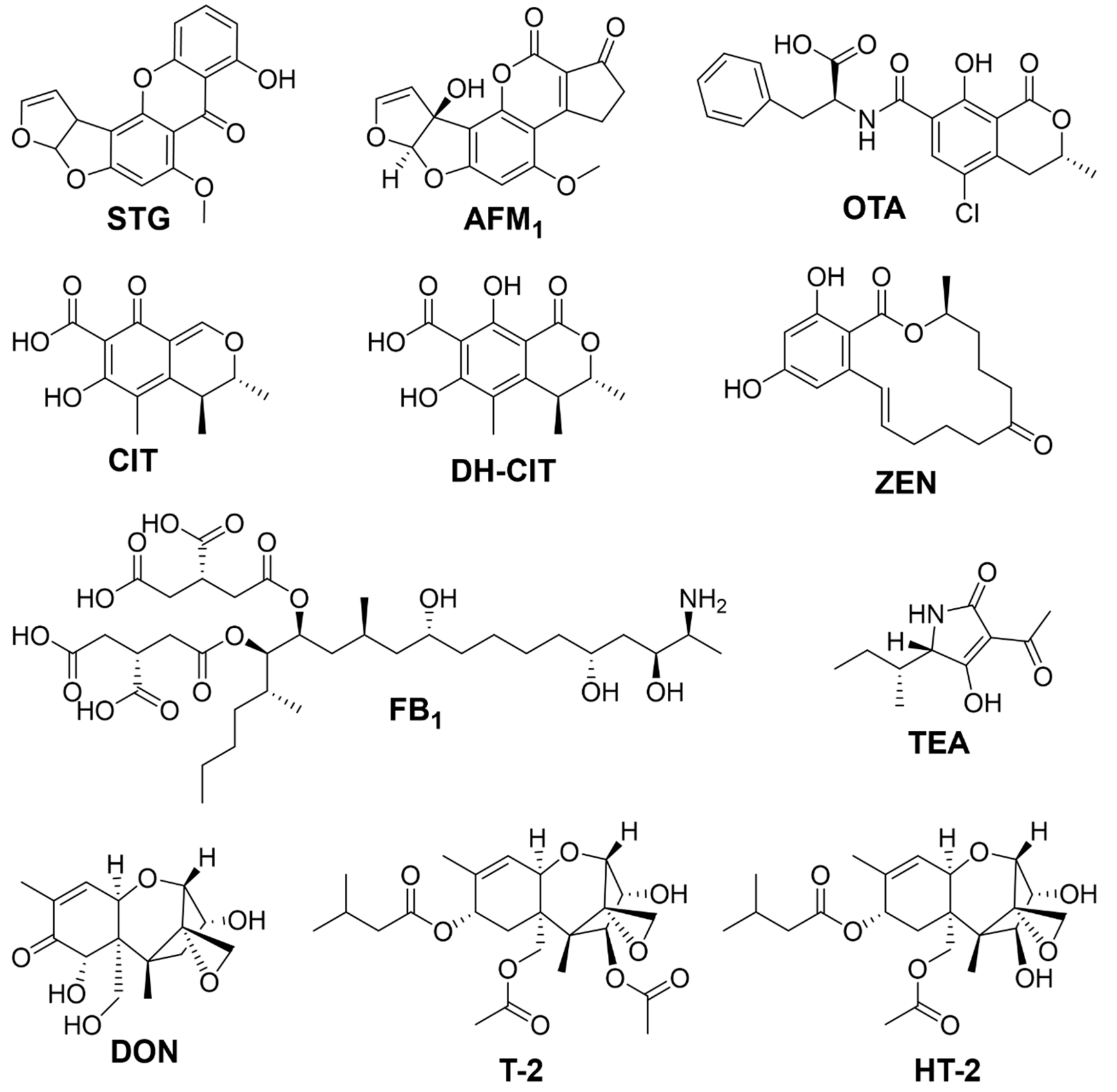

Figure S1 Chemical structures of the 11 mycotoxins, for which matrix effects were studied in urine samples. Sterigmatocystin (STG), ochratoxin A (OTA), citrinin (CIT), dihydrocitrinone (DH-CIT), aflatoxin M_1 (AFM_1), fumonisin B_1 (FB_1), deoxynivalenol (DON), T-2 toxin (T-2), HT-2 toxin (HT-2), zearalenone (ZEN), and tenuazonic acid (TEA).

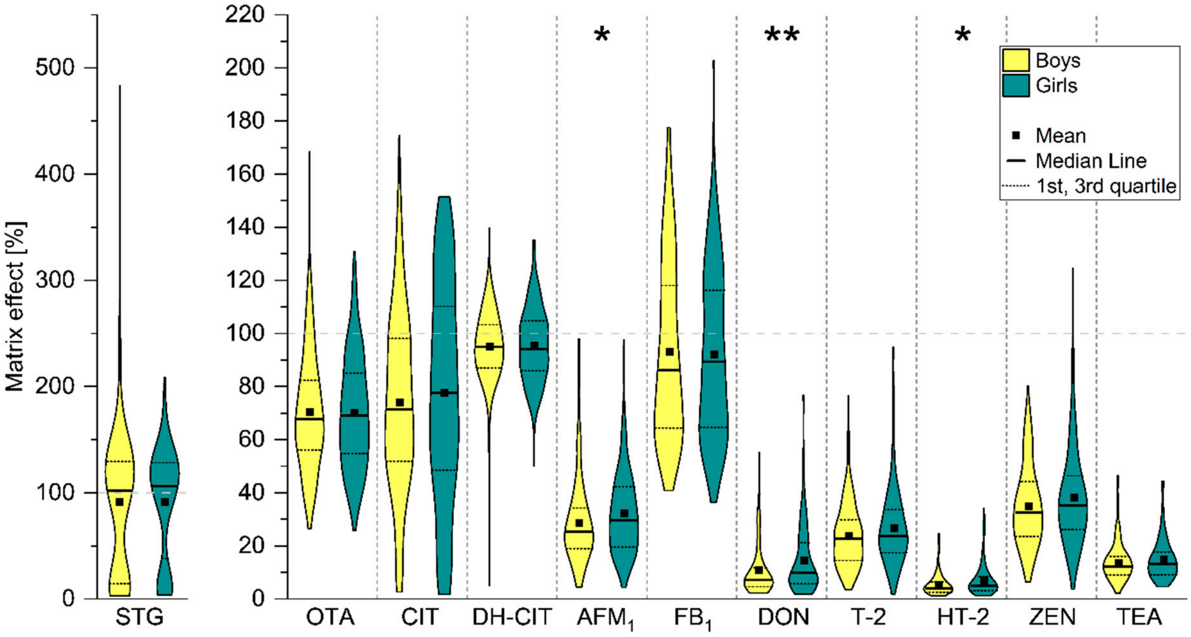

Figure S2 Comparison of matrix effects of sterigmatocystin (STG), ochratoxin A (OTA), citrinin (CIT), dihydrocitrinone (DH-CIT), aflatoxin M_1 (AFM_1), fumonisin B_1 (FB_1), deoxynivalenol (DON), T-2 toxin (T-2), HT-2 toxin (HT-2), zearalenone (ZEN), and tenuazonic acid (TEA) in boys (n = 168) and girls (n = 171) in the Swedish cohort. Violin plots depict the matrix effect distribution within each cohort. Asterisks indicate significantly different median values between both sexes determined by Mann-Whitney test (* $0.05> p >0.01$, ** $0.01> p >0.001$).

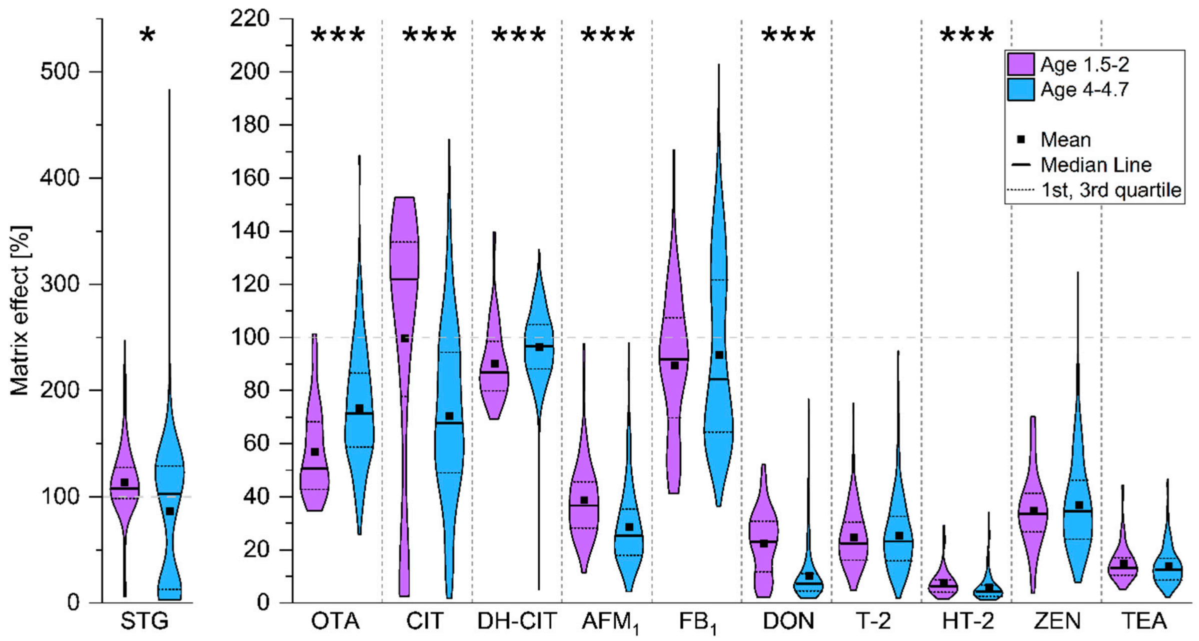

Figure S3 Comparison of matrix effects of sterigmatocystin (STG), ochratoxin A (OTA), citrinin (CIT), dihydrocitrinone (DH-CIT), aflatoxin M_1 (AFM_1), fumonisin B_1 (FB_1), deoxynivalenol (DON), T-2 toxin (T-2), HT-2 toxin (HT-2), zearalenone (ZEN), and tenuazonic acid (TEA) in two age subgroups in the Swedish cohort. First age group ranged from 1.5 to 2 years (n = 64) and second from 4 to 4.7 years (n = 276). Violin plots depict the matrix effect distribution within each cohort. Asterisks indicate significantly different median values between both age groups determined by Mann-Whitney test (* 0.05> p >0.01, *** p <0.001).

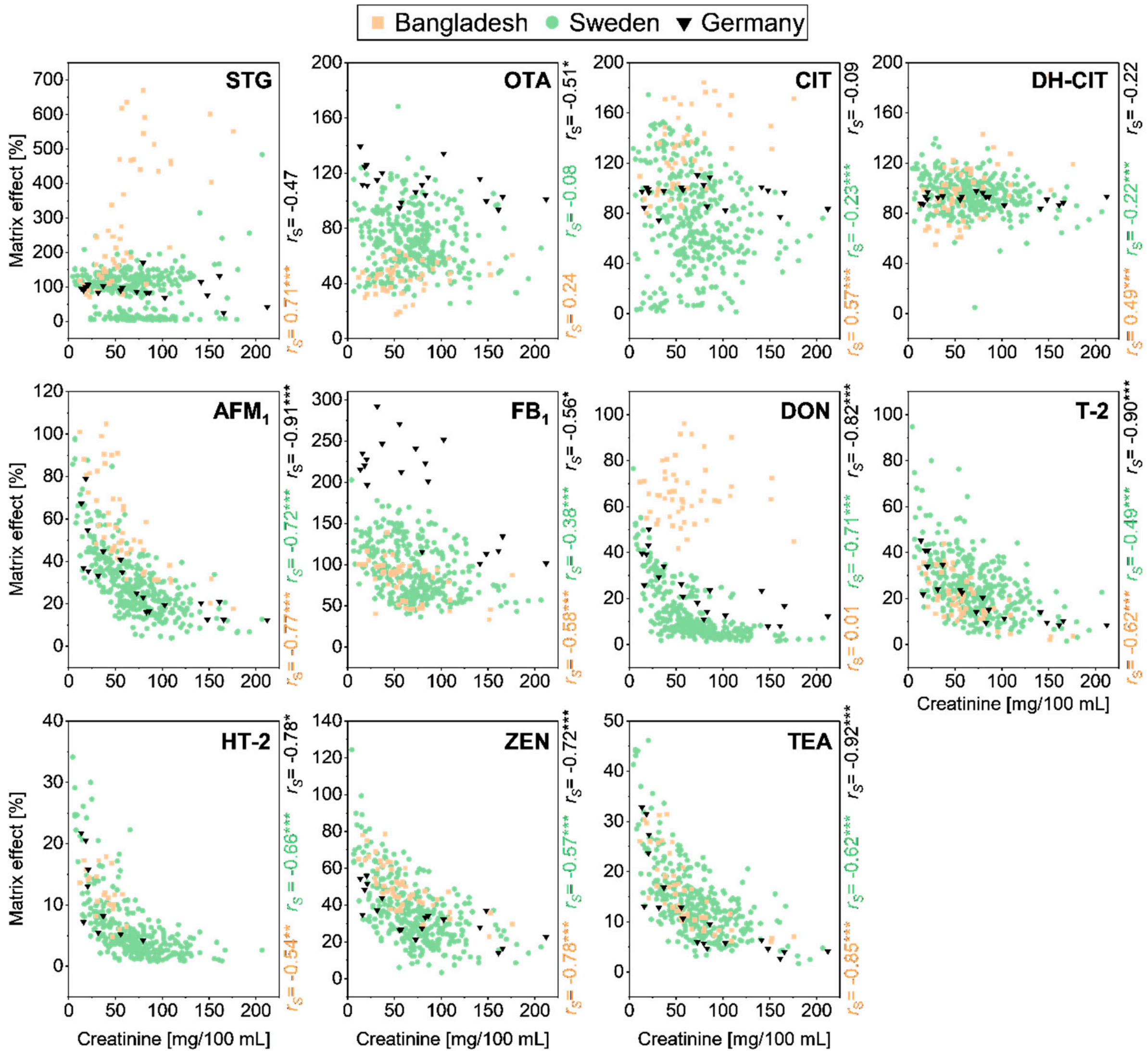

Figure S4 Correlation of matrix effect and creatinine concentration for sterigmatocystin (STG), ochratoxin A (OTA), citrinin (CIT), dihydrocitrinone (DH-CIT), aflatoxin M_1 (AFM_1), fumonisin B_1 (FB_1), deoxynivalenol (DON), T-2 toxin (T-2), HT-2 toxin (HT-2), zearalenone (ZEN), and tenuazonic acid (TEA) in the Bangladeshi (n = 50), Swedish (n = 340), and German (n = 19, from 4 subjects) cohorts. Strength of correlation is indicated by the Spearman correlation coefficient r_S. Asterisks indicate significance of the correlation coefficient (* 0.05> p >0.01, ** 0.01> p >0.001, *** p <0.001).
